# Treatment of Cyanobacterial (Microcystin) Toxicosis Using Oral Cholestyramine: Case Report of a Dog from Montana

**DOI:** 10.3390/toxins5061051

**Published:** 2013-05-29

**Authors:** Kelly A. Rankin, Karen A. Alroy, Raphael M. Kudela, Stori C. Oates, Michael J. Murray, Melissa A. Miller

**Affiliations:** 1 Flathead Animal Clinic, 344 1st Ave. W., Kalispell, MT 59901, USA; E-Mail: kelrankin@gmail.com; 2 Friendship Hospital for Animals, 4105 Brandywine St. NW, Washington, DC 20016, USA; E-Mail: kalroy01@gmail.com; 3 Department of Ocean Sciences, University of California Santa Cruz, A-312 Earth & Marine Sciences Building Santa Cruz, CA 95064, USA; E-Mail: kudela@ucsc.edu; 4 Marine Wildlife Veterinary Care and Research Center, Department of Fish and Game, Office of Spill Prevention and Response, 1451 Shaffer Rd, Santa Cruz, CA 95060, USA; E-Mail: soates@ospr.dfg.ca.gov; 5 Monterey Bay Aquarium, 886 Cannery Row, Monterey, CA 93940, USA; E-Mail: mmurray@mbayaq.org

**Keywords:** acute hepatitis, blue-green algae, cholestyramine, cyanobacteria, hepatotoxin, microsystin, poisoning, silibinin

## Abstract

A two and a half year old spayed female Miniature Australian Shepherd presented to a Montana veterinary clinic with acute onset of anorexia, vomiting and depression. Two days prior, the dog was exposed to an algal bloom in a community lake. Within h, the animal became lethargic and anorexic, and progressed to severe depression and vomiting. A complete blood count and serum chemistry panel suggested acute hepatitis, and a severe coagulopathy was noted clinically. Feces from the affected dog were positive for the cyanobacterial biotoxin, microcystin-LA (217 ppb). The dog was hospitalized for eight days. Supportive therapy consisted of fluids, mucosal protectants, vitamins, antibiotics, and nutritional supplements. On day five of hospitalization, a bile acid sequestrant, cholestyramine, was administered orally. Rapid clinical improvement was noted within 48 h of initiating oral cholestyramine therapy. At 17 days post-exposure the dog was clinically normal, and remained clinically normal at re-check, one year post-exposure. To our knowledge, this is the first report of successful treatment of canine cyanobacterial (microcystin) toxicosis. Untreated microcystin intoxication is commonly fatal, and can result in significant liver damage in surviving animals. The clinical success of this case suggests that oral administration of cholestyramine, in combination with supportive therapy, could significantly reduce hospitalization time, cost-of-care and mortality for microcystin-poisoned animals.

## 1. Introduction

Death from microcystin intoxication has been reported in fish [1,2], birds [2,3], companion animals [4,5,6], livestock [7,8], wildlife [9,10] and humans [11,12], and significant blooms can cause widespread morbidity and mortality [13]. Microcystins are structurally diverse cyclic heptapeptides that are primarily considered as hepatotoxins [14], although the gastrointestinal tract, kidney and other organs are also susceptible to toxin-mediated damage [15]. Human and animal exposure to microcystin typically occurs through ingestion of contaminated water or food, or by licking contaminated fur. 

**Figure 1:**
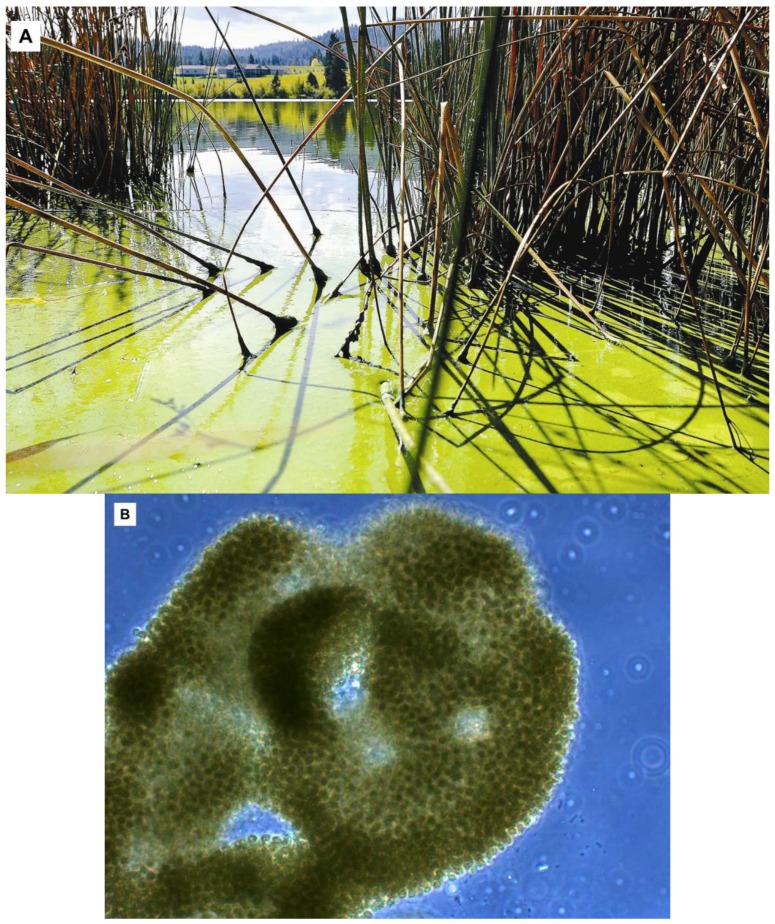
Algal bloom caused by the cyanobacterium *Microcystis aeruginosa* in Middle Foy Lake near Kalispell, Montana in September, 2010. Photograph courtesy of Nate Chute, Daily Inter Lake [16] (**A**); Confirmation of this bloom as *M*. *aeruginosa* microscopically (cell size 2–3 µm) and detection of high concentrations of the cyanobacterial hepatotoxin microcystin-LA in surface scum was performed by scientists at the University of California, Santa Cruz (**B**).

In this case report a two and a half year old 11.6 kg (25.5 lb) spayed female Miniature Australian Shepherd presented on 13 September 2010, to the Flathead Animal Clinic in Kalispell, Montana, with an acute onset of anorexia, vomiting and profound depression. Two days prior to hospitalization, the dog was with her owners at Middle Foy Lake, a small (14 ha (0.05 mi^2^)), shallow lake located near Kalispell, northwest Montana. Middle Foy Lake is located adjacent to a small housing development and is used for recreation and fishing, and it is periodically stocked with rainbow trout. On the day of this visit, Middle Foy Lake contained a prominent, bright green algal surface scum (Figure 1A). The dog walked through this algal scum, and was observed by the owners to be drinking the pond water and licking water and algal scum from her fur coat. Within h of exposure, lethargy and anorexia developed, and then rapidly progressed to severe depression and vomiting. 

## 2. Case Report

At the time of hospitalization, the dog was vomiting, anorexic and depressed. She was afebrile, 38.4 °C (101.2° F), with pink mucus membranes and a capillary refill time of <2 s. Abdominal palpation did not elicit pain, and no evidence of loose stool was noted. A complete blood count and serum chemistry panel revealed lymphopenia, thrombocytopenia and profound elevations of total bilirubin, alanine aminotransferase (ALT) and alkaline phosphatase (ALP) (Table 1). Intravenous fluid therapy (IV) (0.9% sodium chloride) was initiated at 200 mL/h for twenty minutes, and then lowered to a maintenance rate of 30 mL/h (approximately 60 mL/kg/day (27.3 mL/lb/day)). Denosyl (Nutramax Laboratories, Inc., Edgewood, MD, USA), a nutritional supplement containing *S*-Adenosylmethionine (SAM-e) was administered (20 mg/kg (8.8 mg/lb), per os (PO), q 24 h). SAM-e (*S*-adenosylmethionine) is formed from methionine and adenosine triphosphate (ATP). It acts as a methyl group donor to facilitate cellular biochemical reactions and may help reduce cholestasis. Anti-inflammatory and analgesic activity has also been attributed to SAM-e. An attempt to assess clotting parameters was unsuccessful and resulted in severe hemorrhage at the venipuncture site.

**Table 1:** Clinical hematological and serum chemistry parameters in a dog following acute microcystin intoxication and initiation of oral cholestyramine therapy.

	Exposure to cyanobacteria	Presented to clinic *	Declining clinical condition	Initiation of oral cholestyramine therapy	Released to owners for home care	Office recheck	Office recheck *
Timeline of events							
No. days post toxin exposure	0–24 h	2 days	6 days	7 days	10 days	17 days	117 days
No. days of hospitalization	Pre-admission	0 h	4 days	5 days	8 days	Discharged	Discharged
Clinical symptoms ^†^							
Depression	2	3	2	2	1	0	0
Anorexia	2	3	3	3	1	0	0
Vomiting	3	3	3	2	0	0	0
Icterus	0	0	3	3	2	1	0
Abnormal bleeding, hematemesis	0	3	3	3	0	0	0
Complete blood count ^‡^							
WBC (6–17 × 10^3^/μL)	-	10.37	12.45	17.27^ a^	19.33^ a^	8.63	9.6
LYM (1–4.8 × 10^3^/μL)	-	0.77 ^b^	1.89	2.23	2.94	2.66	3.42
NEU (3–12 × 10^3^/μL)	-	8.79	8.94	13.27^ a^	14.83^ a^	4.94	4.92
RBC (5.5–8.5 × 10^6^/μL)	-	7.93	5.35 ^b^	4.85 ^b^	6.5	6.88	8.16
HGB (12–18 g/dL)	-	17.8	11.7 ^b^	10.7 ^b^	15.4	14.8	18.6^ a^
HCT (37%–55%) ^§^	-	51.43	34.58 (33) ^b^	31.64 (31) ^b^	43.40 (42)	44.67	52.72 (53)
PLT (200–500 × 10^3^/μL)	-	29 ^b^	18 ^b^	29 ^b^	116 ^b^	309	215
Reviewer comments		Schistocytes	Schistocytes codocytes echinocytes				
Serum chemistry panel ^‡^							
ALB (2.5–4.4 g/dL)	-	4	1.8 ^b^	1.7 ^b^	2.9	2.9	4.3
ALP (20–150 U/L)	-	294 ^a^	621 ^a^	465 ^a^	472 ^a^	290 ^a^	150
ALT (10–118 U/L)	-	NM	8205 ^a^	5486 ^a^	3640 ^a^	835 ^a^	42
TOTAL BILI (0.1–0.6 mg/dL)	-	7.3 ^a^	12.3 ^a^	13.6 ^a^	8.0 ^a^	1.8 ^a^	0.2
BUN (5–31 mg/dL)	-	25	31	39 ^a^	14	9	26
CRE (0.3–1.4 mg/dL)	-	ICT	1.7 ^a^	1.7 ^a^	1.3	1.2	1.1
PHOS (2.9–6.6 mg/dL)	-	5	6.3	6.2	4.7	4.5	5.2
GLU (60–110 mg/dL)	-	97	87	88	93	92	104
CA++ (8.6–11.8 mg/dL)	-	11.1	8.3 ^b^	8.4 ^b^	10.2	10.5	11.2
NA+ (138–160 mmol/L)	-	139	142	145	147	145	143
K+ (3.7–5.8 mmol/L)	-	4.3	4.2	4	4.2	4.6	4.6
CL− (110–118 mmol/L)	-	-	117	122 ^a^	110	115	-
AST (1–37 U/L)	-	-	1419 ^a^	367 ^a^	160 ^a^	67 ^a^	-
CK (25–467 U/L)	-	-	110	147	61	81	-
CHOLESTEROL (126–359 mg/dL)	-	-	94 ^b^	79 ^b^	110 ^b^	147	-
Hemolyzed	-	2+	-	-	-	-	0
Lipemic	-	0	-	-	-	-	1
Icteric	-	2+	-	-	-	-	0

* = blood samples analyzed in-clinic with VetScan HM5 Hematology Analyzer; all other blood chemistry values were determined by Kalispell Regional Medical Center Laboratory; † = Categories defined as: 0 = not detected; 1 = mild; 2 = moderate; 3 = severe; ‡ = values in parenthesis represent the normal range; § = Hematocrit values presented without parentheses are machine-derived, while values in parentheses were determined by hand for the same sample; a = above normal reference values; b = below normal reference values; NM = no measurement, values too high for machine to read; ICT = value supressed, results affected by >10% interference from icterus; - = information not available.

Over the next few days the dog’s condition continued to deteriorate. On the third day of hospitalization (five days post-exposure), the mucus membranes, sclera, and pinnae became markedly icteric. Dark yellow urine and tarry stools were also noted. The IV fluid was supplemented with 20–32 m Eq/L of potassium chloride (KCl) and 5% Dextrose to correct for enteric potassium loss and continuing anorexia. Results from an IDEXX clotting profile came back as non-diagnostic due to a clotted sample. It was suspected that either the sample was roughly handled post-sampling, or that the animal was in a hypercoaguable state consistent with disseminated intravascular coagulation (DIC).

On day four of hospitalization (six days post-exposure), repeated hematology and serum chemistry assays revealed progressive and severe elevations of total bilirubin, ALT, and ALP as well as high levels of aspartate aminotransferase (AST), reflective of severe hepatocellular injury and cholestasis. (Table 1). In addition, the dog was hypocalcemic, hypoproteinemic, hypoalbuminemic and hypocholesterolemic, with a mild elevation of serum creatinine. The serum was visibly icteric, with worsening thrombocytopenia (18,000 platelets per µL) and mild anemia. Occasional, codocytes, acanthocytes, and schistocytes were observed on blood smears, indicating liver disease and DIC. The dog remained severely depressed and anorexic and she continued to vomit and produce dark yellow/brown colored urine. 

Based on these discouraging findings, additional supportive measures were added, including vitamin B complex supplementation to help facilitate lipid metabolism (B1 25 mg, B2 4 mg, B3 25 mg, B6 10 mg, B12 10 μg, IV, q 24 h), famotidine to minimize gastric ulceration and bleeding (0.5 mg/kg (0.23 mg/lb), SC, q 24 h), and procaine penicillin G to prevent septicemia resulting from decreased hepatic bacterial clearance (PPG 300,000 U/kg (136,364 u/lb), IM, q 24 h). The IV fluid infusion rate was increased to 60 mL/h to promote diuresis (120 mL/kg/day (54.5 mL/lb/day)) and oral SAM-e supplementation was continued. A considerable amount of hemorrhage was observed after intramuscular (IM) administration of PPG, so IV administration of penicillin G potassium was substituted (35,000 u/kg (15,910 u/lb), IV, q 24 h). Urine and blood samples were submitted for polymerase chain reaction (PCR) detection of *Leptospira* spp. Despite a somewhat brighter disposition, the dog remained icteric and continued to vomit during day five of hospitalization (seven days post-exposure). She developed hematemesis, and the color of vomitus ranged from bright red to brownish-black, indicative of a high content of undigested (red) and partially digested (black) blood within the gastric lumen. Hematology and serum chemistry assays revealed progressive increases in both total bilirubin and Blood Urea Nitrogen (BUN). The concentrations of ALT and ALP remained markedly elevated, although absolute values had decreased relative to the previous blood sample (Table 1). Also noted were continued mild anemia, mild leukocytosis and neutrophilia. Serum platelets had increased to 29,000 per µL, but still well below normal levels. The dog remained mildly hypocalcemic, hypoproteinemic, hypoalbuminemic, and hypocholesterolemic, with a mild elevation of serum creatinine. Based on these findings, the dog’s IV fluid infusion rate was returned to 30 mL/h.

On day 5, after telephone consultation with the study co-authors, treatment with cholestyramine (Cholestyramine Light Packet (Sandoz, Princeton, NJ, USA), 172 mg/kg (78.4 mg/lb) mixed with water, PO, q 24 h) was initiated. This medication was added to the treatment regimen based on a previously published report that demonstrated the ability of oral cholestyramine to bind enteric cyanotoxins (microcystin-LR) in laboratory rats, thereby reducing toxin-related morbidity and mortality [17]. 

During the next two days, slow but progressive clinical improvement was observed. On day six of hospitalization, the dog’s temperament was bright, but she remained icteric and was only mildly interested in food. By the following day, vomiting had ceased, and the mucous membranes and sclera were pinker than on previous days. All medications were continued as before, with the addition of Denamarin (Pfizer Animal Health, New York, NY, USA) on day six (SAM-e 20 mg/kg (8.8 mg/lb) plus silibinin 2 mg/kg (0.94 mg/lb), PO, q 24 h) and supplementary thiamine (8.6 mg/kg (4.0 mg/lb), IV, q 24 h) on day seven to address potential deficiencies due to continued anorexia. Additionally, on day six of hospitalization (day 8 post-exposure), fresh feces from the affected dog were submitted to the University of California, Santa Cruz for cyanotoxin screening via liquid chromatography/mass spectrophometry (LC/MS). Surface scum collected from the ongoing algal bloom at Middle Foy Lake was also submitted for microscopic examination and LC/MS testing. 

By day seven of hospitalization, the icterus was resolving, vomiting had ceased and the dog was eating small amounts of food. The *Leptospira* spp. PCR results were negative. A complete blood count and serum chemistry panel revealed significant improvements in total bilirubin, ALT and ALP concentrations and an increased platelet count (Table 1). On day eight, intravenous fluids were discontinued and the animal was discharged with instructions to rest and recheck bloodwork in 5–7 days. Oral cholestyramine and Denamarin (SAM-e and silibinin) therapy were continued at home at the same dosages for 14 days following hospital discharge. Additionally, famotidine (0.4 mg/kg (0.2 mg/lb), PO, q 24 h), amoxicillin (20 mg/kg (9.1 mg/lb), PO, q 12 h), and an iron-rich vitamin supplement (Lixotinic, 6 mL, PO, q 24 h) were prescribed. For the subsequent 5–6 weeks, the dog was maintained on a diet of Hills l/d, a prescription diet formulated for hepatic support. 

The dog presented to the clinic one week later for follow-up evaluation. While slight icterus was still detectable, all other clinical abnormalities had resolved, with no vomiting noted since leaving the hospital. The platelet count had returned to normal, and the total bilirubin and liver enzyme values were greatly reduced. During a recheck examination four months post-exposure, the complete blood count and serum chemistry panel findings were unremarkable and no clinical abnormalities were noted. 

Surface scum collected by the study co-authors from Middle Foy Lake during the bloom event contained 38,627 ppb microcystin-LA, and large numbers of cyanobacteria compatible with *Microcystis aeruginosa* were observed in this same sample via light microscopy. Feces from the exposed dog collected eight days post-exposure also contained 217 ppb of microcystin-LA. The microcystin congeners -LR, -RR, and -YR were not detected at appreciable levels in these samples, nor was there any indication of other microcystin congeners, based on total ion chromatograms. After the treating DVM (Rankin) reported her findings to local public health authorities, they issued a press release regarding potential animal and human health risks associated with water contact at the lake. This notification also catalyzed efforts to provide ongoing surveillance, and mitigate toxic cyanobacterial blooms at this site by the local community. 

## 3. Discussion

To the author’s knowledge, this is the first description of successful treatment of biochemically-confirmed cyanobacterial (microcystin-LA) toxicosis in a dog. Based on published reports [4,5,6,8,18], most affected animals die or are euthanized due to the severity of their disease, a perceived lack of efficacious treatment options and the high cost of prolonged supportive care. However, our results describing expedited recovery of a microcystin-poisoned dog, as well as prior laboratory studies, suggest that oral cholestyramine administration could reduce treatment costs, shorten recovery times and enhance patient survival. Toxin removal from the gastrointestinal tract and aggressive supportive care, including IV fluid therapy are core therapeutic measures. Other critical steps include preventing access to contaminated water and washing exposed animals to remove residual toxin from their fur [19]. Supplementation with B vitamins may also help support liver function, as well as the induction of emesis or oral administration of activated charcoal to minimize microcystin absorption and enterohepatic recirculation [5]. 

The dog reported in this study exhibited dark tarry stools and abnormal bleeding. It is unknown which of the following were the primary contributors to the observed coagulopathy: severe hepatic damage leading to depletion of hepatogenic clotting factors, vascular mural or endothelial damage, cyanobacterial lipopolysaccharide endotoxicosis, impaired function of clotting factors, red blood cells or platelets, clinically confirmed thrombocytopenia, secondary shock and/or disseminated intravascular coagulation. For severe cyanobacterial intoxication, blood, plasma or platelet transfusions may be necessary [19]. A multi-modal therapeutic approach was utilized in this case, including IV fluids, mucosal protectants, vitamins, antibiotics, nutritional supplementation and oral application of cholestyramine and silibinin; two therapeutic agents with reported hepatoprotective properties. To the author’s knowledge, no prior studies have described the clinical application of these two compounds for treatment of microcystin intoxication in naturally occurring cases.

Cholestyramine is a non-digestible ion exchange resin that binds bile in the gastrointestinal tract and prevents enterohepatic recirculation of bile acids and associated bound substances. In humans it has been used to treat hypercholesterolemia and cholestatic pruritis (from 50 mg/kg (22.7 mg/lb) to 200 mg/kg (91 mg/lb), PO, q 24h) [20]. It has also been used to treat humans with Possible Estuarine-Associated Syndrome (112 mg/kg (51 mg/lb) PO, q 6 h), a syndrome caused by ingestion of toxins from the dinoflagellate (*Pfiesteria*) [21]. Cholestyramine can bind >99% of microcystin-LR [17] *in vitro*, and *in vivo* studies in rats have confirmed that cholestyramine binds microcystin-LR in the gastrointestinal lumen. When intra-intestinal cholestyramine (175–550 mg/kg (80–250 mg/lb)) was administered immediately following experimental intra-intestinal administration of microcystin-LR (2.5–5 mg/kg (1.1–2.3 mg/lb)) in rats, only mild hepatocyte rounding was observed, in contrast to severe hepatocyte degeneration and necrosis in rats that received the same dose of microcystin without cholestyramine [17]. 

In humans, oral administration of high doses of cholestyramine (200–250 mg/kg (91–114 mg/lb), PO, q 24 h) is associated with bloating, constipation, nausea and flatulence [21]. Cholestyramine may also impair intestinal absorption of other medications. To minimize this effect, it should be administered one hour before, or 4 h following meals or administration of other oral medications [21]. The microcystin-poisoned dog was treated at a lower dose (172 mg/kg (78 mg/lb), PO, q 24 h) and no new clinical abnormalities were observed after cholestyramine administration was initiated. 

The dog was also treated with an oral supplement (Denamarin, Sigma Aldrich Corporation, St. Louis, MO, USA) that contains 225 mg of SAM-e (an antioxidant) and 24 mg of silibinin. Silibinin is the major active ingredient of milk thistle (*Silybum marianum*) extract, (also known as Silymarin), a mixture of flavonolignans, including silibinin A and B, isosilibinin A and B, silicristin and silidianin. *In vitro* and *in vivo* studies suggest that silymarin and silibinin have antihepatotoxic properties [22,23,24,25], and silymarin may help neutralize the hepatotoxic effects of ethanol, acetaminophen, and amatoxins produced by toxic mushrooms, including *Amanita phalloides* [24]. Lakshamana Rao *et al.* [25] demonstrated that intraperitoneal pre-treatment with silymarin (400 mg/kg (181 mg/lb) IP) in mice is protective against microcystin-LR toxicity. Intraperitoneal (IP) administration of microcystin-LR (0.1 mg/kg (0.05 mg/lb)) alone resulted in 100% mortality in mice, while IP treatment with silymarin at 3 and 24 h before microcystin administration resulted in 0% mortality [25]. Mereish *et al.* [22] also reported that intraperitoneal pre-treatment with silymarin (150–500 mg/kg (68–227 mg/lb), IP) in rats reduced mortality due to microcystin-LR toxicity, but pre-treatment was less efficacious at lower dosages (150–350 mg/kg (68–160 mg/lb), IP). 

In contrast with IP therapy, oral pre-treatment with silymarin, even at high doses (500 mg/kg (227 mg/lb), PO), did not prevent microcystin toxicity in rats, with 100% mortality observed [23]. The medical literature also documents poor bioavailability of oral silymarin in humans [24]. To date, studies examining the clinical efficacy of silibinin and silymarin have been difficult to interpret due small sample sizes and variations in research objectives, dosage, routes of administration and extract composition [24]. In addition, the dosage of oral silibinin administered in this case (2 mg/kg (0.94 mg/lb), PO, q 24 h) was much lower and less frequent than those from published reports. While silibinin may have contributed to this dog’s recovery in some capacity, the conservative dosage and oral route of administration were unlikely to have strongly influenced clinical outcome, based on published findings in other species. 

Although a controlled study was not possible, the temporal association between initiation of oral cholestyramine therapy and the onset and speed of clinical and hematological recovery is striking in this case. Cholestyramine therapy was initiated six days post-exposure after a long period of worsening, or at best, static condition. Notable clinical improvement was appreciated after 48 h and by the third day of cholestyramine therapy, significant improvements in platelet counts, hematocrit and total bilirubin were also detected (Table 1). This dog went on to make a full clinical recovery, with normalization of all blood values, and she remains clinically normal one year post-exposure. Experimental studies in combination with these clinical findings appear to support the efficacy of cholestyramine as a treatment of microcystin intoxication. Combination therapy of both cholestyramine and silibinin might further improve clinical outcome, however, higher silibinin dosages and/or parenteral administration would probably be necessary. More robust studies of the efficacy of cholestyramine and/or silibinin for treating human and animal intoxication by microcystin and other cyanotoxins are warranted.

Unfortunately, rapid, inexpensive, and widely accessible assays are not currently available to facilitate rapid diagnosis and treatment of cyanobacterial intoxication. Many commercial diagnostic laboratories offer few or no diagnostic tests for cyanotoxin detection, so clinicians wishing to perform cyanotoxin testing should consult with federal, state and academic toxicology laboratories. The gold standard tests for toxin identification and characterization are currently LC/MS, gas chromatography/mass spectrophotometry, or liquid chromatography combined with tandem spray mass spectrophotometry [12]. Cyanotoxins can be detected in liver, bile, feces, urine, and serum, but samples should be kept at ≤4 °C (39.2° F) if stored for prolonged periods prior to testing [9,12,26]. Microcystins are relatively stable, once formed, and have been detected in tissues stored for >10 years at −70 °C [12]. Based on limited testing of known-positive otters, liver, bile and feces appear to be optimal samples for postmortem microcystin detection, while feces, digesta and vomitus could be used for screening live animals. However, based on biochemical tests of a number of sea otters that were microcystin-positive on LCMS/MS tests of liver, feces and/or bile, serum proved to be a comparatively insensitive sample matrix that often yielded false negative results [27]. Because of this insight regarding test performance and due to cost constraints, serum testing for microcystin was not attempted in this case. A publication detailing test results in different sample matrices for sea otters is in preparation. Bioassays have also been used to confirm diagnosis by injecting laboratory mice with suspect material (1.0 mL IP) [5,7]. 

In the current study, the presence of a toxic cyanobacterial bloom at Middle Foy Lake was confirmed both microscopically and biochemically (Figure 1B). Both surface scum from the lake and feces from the exposed dog tested positive for microcystin-LA at 38,627 and 217 ppb, respectively. The concentration of microcystin-LA detected in algal scum from Middle Foy Lake was nearly 4000 times the World Health Organization advisory level for recreational exposure, and almost 40,000 times the established limit for microcystin contamination of finished drinking water [14]. In humans, microcystin exposure can cause dermatitis, ophthalmitis, ataxia, acute gastroenteritis and, rarely, severe hepatic injury or death [28]. Chronic exposure to cyanobacterial toxins in humans has been linked to development of primary liver cancer and neurodegenerative disease [29,30]. 

Through rapid recognition, treatment and reporting of cyanotoxin poisoning cases, veterinarians can help prevent animal deaths, limit human and animal exposure, and promote mitigation of cyanobacterial blooms in nutrient-impaired water bodies. Because microcystins are regulated pollutants that can cause illness and death in humans, opportunities for catalyzing environmental mitigation are substantial. At present there is a significant unmet need for simple and inexpensive diagnostic tests to expedite diagnosis and treatment for humans, domestic animals and wildlife that have been poisoned by cyanotoxins. Once these tests are more widely available, we predict that case recognition will increase dramatically throughout North America and worldwide. In addition, both the quality and success of clinical care, and efforts to control and prevent cyanobacterial blooms can be expected to make significant advances.

## 4. Conclusions

This manuscript provides the first report of successful treatment of canine cyanobacterial (microcystin) toxicosis. Untreated microcystin intoxication is commonly fatal. The clinical success of this case suggests that administration of cholestyramine, in combination with supportive care could significantly reduce morbidity and mortality due to microcystin intoxication. In addition to improving survival rates for affected animals, oral cholestyramine therapy may also shorten the clinical course of microcystin intoxication, thus decreasing veterinary costs and ensuring that more animals receive veterinary care post-exposure. Veterinarians can help prevent additional animal deaths and expedite mitigation of cyanobacterial blooms by reporting suspect and confirmed cases to local public health and water quality officials. 
